# Social inequalities in the risk of giving birth to a small for gestational age child in Sweden 2010–16: a cross-sectional study adopting an intersectional approach

**DOI:** 10.1093/eurpub/ckad184

**Published:** 2023-10-25

**Authors:** Sten Axelsson Fisk, Jesper Alex-Petersen, Mikael Rostila, Can Liu, Sol Pia Juárez

**Affiliations:** Department of Clinical Sciences Lund, Obstetrics and Gynaecology, Lund University, Lund, BMC C14. Lund, 22185, Sweden; Department of Obstetrics and Gynaecology, Ystad Hospital, Ystad, Sweden; Department of Obstetrics and Gynaecology, Ystad Hospital, Ystad, Sweden; Department of Public Health Sciences, Stockholm University, Stockholm, Sweden; Centre for Health Equity Studies (CHESS), Stockholm University/Karolinska Institutet, Stockholm, Sweden; Department of Neurobiology, Care Sciences and Society (NVS), Aging Research Center (ARC), Karolinska Institutet/Stockholm University, Stockholm, Sweden; Department of Public Health Sciences, Stockholm University, Stockholm, Sweden; Centre for Health Equity Studies (CHESS), Stockholm University/Karolinska Institutet, Stockholm, Sweden; Clinical Epidemiology Division, Department of Medicine, Solna, Karolinska Institutet, Stockholm, Sweden; Department of Public Health Sciences, Stockholm University, Stockholm, Sweden; Centre for Health Equity Studies (CHESS), Stockholm University/Karolinska Institutet, Stockholm, Sweden

## Abstract

**Background:**

Well-established associations exist between the risk of small for gestational age (SGA) and unidimensional sociodemographic factors. We investigated social inequalities in SGA risk and adopted an intersectional approach that simultaneously considers different social categories. By doing so, we could assess heterogeneities in SGA risk within unidimensional sociodemographic categories.

**Methods:**

We included all live 679 694 singleton births in Sweden between 2010 and 2016. The outcome was SGA, and the exposures were age, maternal educational level, dichotomous migration status and civil status. Thirty-six possible combinations of these factors constituted the exposure in an intersectional model. We present odds ratios (ORs) with 95% confidence intervals (95% CIs) and the area under the receiver operating characteristic curve (AUC)—a measurement of discriminatory accuracy (i.e. the ability to discriminate the babies born SGA from those who are not).

**Results:**

Women with low education and women born outside Sweden had ORs of 1.46 (95% CI 1.38–1.54) and 1.50 (95% CI 1.43–1.56) in unidimensional analyses, respectively. Among women aged under 25 with low education who were born outside Sweden and unmarried, the highest OR was 3.06 (2.59–3.63). The discriminatory accuracy was low for both the unidimensional model that included all sociodemographic factors (AUC 0. 563) and the intersectional model (AUC 0.571).

**Conclusions:**

The intersectional approach revealed a complex sociodemographic pattern of SGA risk. Sociodemographic factors have a low accuracy in identifying SGA at the individual level, even when quantifying their multi-dimensional intersections. This cautions against interventions targeted to individuals belonging to socially defined groups to reduce social inequalities in SGA risk.

## Introduction

Reducing social inequalities in perinatal health outcomes constitutes a central part of Sweden’s public health strategy to eliminate preventable health disparities within a generation.^1^ Many studies reporting social inequalities in risk of small for gestational age (SGA) adopt a unidimensional methodology that considers one social dimension at the time.^2^^,^^3^ The unidimensional approach to study educational disparities in risk of SGA would typically include adjustment for age, country of birth, civil status and other information deemed relevant by the researcher. Such multiple adjustment approaches fail to capture the heterogeneity within unidimensional categories.^4^ Despite the limitations, unidimensional approach-based findings have been used for making perinatal medical interventions on women belonging to social groups defined in a unidimensional way in regional^5^ or national guidelines in Sweden.^6^ Meanwhile, the need for strengthening the scientific basis for such individual-targeted guidelines remains. Over the last decade, intersectional approaches have been introduced in public health research^7–9^ as one way to address the complex interaction between social and demographic factors. For example, to be born outside Sweden may imply a high risk of SGA for a woman with a low social position, whereas the risk is not necessarily as high for a foreign-born woman with a higher social position. As a broad theory with diverging stances towards the use of social categories in public health, intersectionality can be divided into categorical and anti-categorical intersectionality.^10^ Anti-categorical intersectionality considers social life too complex for simplifications represented by the traditional use of social categories in quantitative research. From this perspective, the use of social categories such as ‘low socioeconomic status’, ‘immigrant’ or ‘unmarried’ by the research community and authorities can contribute to the reproduction of differences between groups. Categorical intersectionality, on the other hand, claims that even imperfect social categories are necessary to monitor social inequalities in health and provide insights into the heterogeneities within unidimensional social categories. To determine if an anti-categorical or categorical approach is most appropriate in a given context for a given outcome, statistical methods should be used that not only present the average differences between the social categories but also quantify the discriminatory accuracy (DA), i.e. how well a social risk factor can separate individuals who will experience an outcome from those that will not.^8^ In the case where the DA is high, the categorical intersectionality is supported since the social categories can presumably be used to target health inequalities. The finding of a low DA, on the other hand, could motivate an anti-categorical stance since in that case the social categorization only explains a minor proportion of health variation. As such, the finding of a low DA implies that targeted interventions on the individual level based on selected social factors may be inefficient and introduce unnecessary stigmatization.

Improved detection of SGA pregnancies is of strategic importance to reduce rates of stillbirth.^11^ In Sweden, SGA pregnancies are uniformly defined as a birthweight below −2 SD, based on 759 ultrasonographic estimations of fetal birthweights in 86 uncomplicated singleton pregnancies from four Scandinavian centres. This SGA definition does not consider demographic factors.^12^

The aim of this study is to examine social inequalities in SGA adopting an intersectional approach. We consider combinations of age, education status, civil status and country of birth simultaneously to elucidate heterogeneity in risk of SGA within these unidimensional social categories.

## Methods

### Data sources and study population

This cross-sectional study is based on data from the Swedish Medical Birth Register (MBR), which contains information from antenatal care and delivery wards to report maternal and child health information, for example, birthweight, maternal body mass index (BMI), smoking habits and comorbidities. Coverage of the MBR is estimated to be 97–99% during the last 20 years.^13^ Sociodemographic data on education, income and civil status are derived from Longitudinal Integrated Database for Health Insurance and Labor Market Studies^14^ and country of birth is derived from the Swedish Total Population Register.^15^

**Figure 1:**
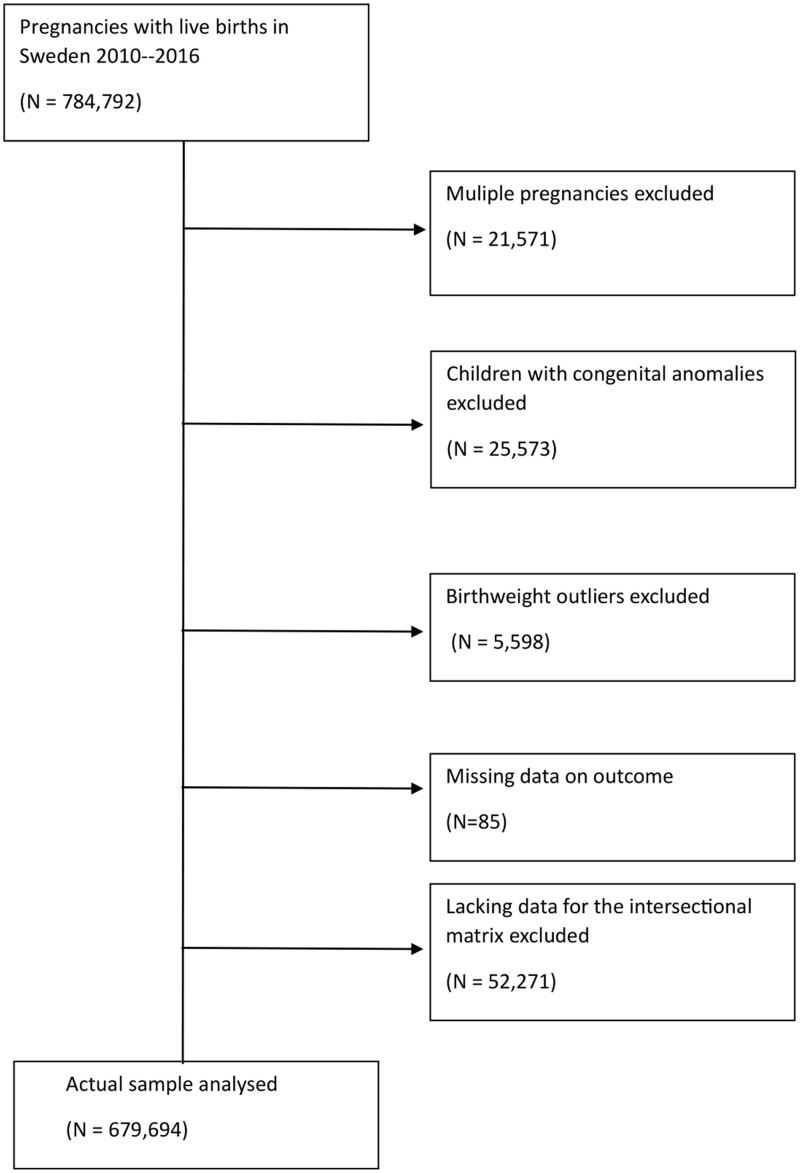
Flow chart showing the selection of the study population

Of 784 792 live birth pregnancies in Sweden between 2010 and 2016, we excluded multiple pregnancies (*n* = 21 571), pregnancies with any malformation diagnoses (*n* = 25 573) and pregnancies with birthweights out of range (*n* = 5,598) defined following the threshold published by Källén.^16^ Finally, 85 of the pregnancies with missing information on the outcome and 52 271 (7.1% of pregnancies before this step of exclusion) pregnancies with missing information on any of the sociodemographic exposure variables were also excluded. This results in a final study population of 679 694 singleton pregnancies with live births, plausible birthweights and without missing information. The flow chart of the selection of the study population is shown in figure 1.

### Variables

SGA is defined according to clinical routines in Sweden as weighing less than −2 SD compared with expected birthweight accounting for gestational age and gender, based on growth curves recommended by Marsál et al.^12^

Maternal age is categorized into three groups: women aged ≤25, women 26–35 years old and women aged ≥36 years at the time of delivery. Education status was categorized into three categories: low (primary education, ≤9 years of education), middle (secondary education, 10–14 years of education) and high (more than 2 years of postsecondary education, >14 years of education). Migration status is categorized into people born in Sweden and immigrants, i.e. people born outside Sweden. Civil status is dichotomously categorized into people being married or living in registered partnership in one category and unmarried, divorced and widowed people in another category. These variables were then used to create the intersectional matrix.

### Statistical analysis

In the first step, we present the distribution of cases of SGA in the different sociodemographic strata. After that, we perform multiple logistic regression analyses in two models and report odds ratios (ORs) and their 95% confidence intervals (CIs). In the first model, we include all the unidimensional sociodemographic variables assessed in this study: age, education status, migration status and civil status. In model 2, we create an intersectional matrix that consists of all unique combinations of these variables, resulting in 36 (3×3×2×2) intersectional strata that is the only exposure variable in this model. Since each unique combination exists in the matrix any potential intersectional interaction between the included variables will lead to improved performance in model 2 compared with model 1.

Following recommendations by Pepe et al.,^17^ we pay special attention to the calculation of the area under the receiver operating characteristic curve (AUC) for both models, as a measurement of DA. The AUC value takes both sensitivity and specificity into account and is a measure of how accurately a model can separate individuals who will have an SGA child from those who will not. The AUC can take a value between 0.5, which implies that the model does not provide more information than flipping a coin, and 1.0, which means that the model perfectly identifies SGA cases. The following cut-off values were adopted as proposed in a previous study: (i) ‘absent or very small’ (AUC = 0.5–0.6), (ii) ‘moderate’ (AUC >0.6 to ≤0.7), (iii) ‘large’ (AUC >0.7 to ≤0.8) and (iv) ‘very large’ (AUC >0.8).^18^

An increase in AUC between model 2 (model with 36 intersectional strata) and 1 (unidimensional model with all sociodemographic variables) indicates that intersectional interaction is occurring, whereas overlapping CIs for the AUC values of these models imply that interaction is absent. Since we use logistic regression analysis, we assess the presence of interaction in the multiplicative scale.^7^

All analyses are performed using STATA^®^ version MP 15.1 (StataCorp, College Station, TX).

### Ethics approval

This investigation is included in the research program Studies of Migration and Social Determinants of Health. This study includes registered data containing sensitive information on human subjects and was approved by Regional Ethical Review Board of Stockholm (decision no. 2017/716-31).

## Results

Table 1 describes the distribution of SGA across sociodemographic groups. In our population, 1.76% of the pregnancies had an SGA child. SGA occurred more frequently among women younger than 26 years and among women 36 years or older compared with women aged 26–35 years. There was an educational gradient where women with higher education had a lower risk of SGA. Immigrants had higher rates of SGA compared with women born in Sweden. Unmarried women had slightly higher rates of SGA compared with married ones.

**Table 1:** Descriptive statistics and frequency of SGA among 679 694 women giving birth to term singleton babies between 2010 and 2016 in Sweden

	Not SGA	SGA
	*n* (%)	*n* (%)
Age		
≤25	117 282 (98.0)	2438 (2.0)
25–35	435 559 (98.4)	7266 (1.6)
≥36	114 909 (98.1)	1724 (1.9)
Education		
Low	76 335 (97.4)	2035 (2.6)
Middle	247 435 (98.2)	4441 (1.8)
High	343 980 (98.4)	5468 (1.6)
Migration status		
Immigrant	137 489 (97.6)	3378 (2.4)
Born in Sweden	530 261 (98.4)	8566 (1.6)
Civil status		
Married	301 539 (98.3)	5169 (1.7)
Not married	366 539 (98.2)	6775 (1.8)

Since the prevalence of SGA was low, ORs can be interpreted as relative risks. ORs and AUC value from model 1 are presented in table 2. In model 1, which includes all sociodemographic factors, OR for women younger than 26 years was 1.10 (95% CI 1.05–1.16) and women older than 35 years had OR of 1.17 (95% CI 1.11–1.22) compared with women aged 26–35. Women with low education had the highest OR of 1.46 (95% CI 1.38–1.54) while women with middle education had OR of 1.10 (95% CI 1.05–1.14) compared with the reference group of women with high education. Immigrants had OR of 1.50 (95% CI 1.43–1.56) compared with women born in Sweden. Unmarried women had OR of 1.16 (95% CI 1.11–1.20) compared with married women.

**Table 2:** ORs and 95% CIs from model 1 and AUC values with 95% CIs from model 1 and 2, results from logistic regression with unidimensional social risk factors as explanatory variables and SGA as the outcome

	Model 1	Model 2
Variable	OR (95% CI)	Intersectional
Age		ORs from Model 2 in table 3
≤25	1.10 (1.05–1.16)	
25–35	Ref	
≥36	1.17, (1.11–1.22)	
Education (years)		
Low	1.46 (1.38–1.54)	
Middle	1.10 (1.05–1.14)	
High	Ref	
Migration status		
Born in Sweden	Ref	
Immigrant	1.50 (1.43–1.56)	
Civil status		
Married	Ref	
Not married	1.16 (1.11–1.20)	
AUC (95% CI)	0.567 (0.562–0.573)	0.571 (0.565–0.576)

Note: Analyses performed among 679 694 women with singleton pregnancies giving birth in Sweden between 2010 and 2016.

ORs from model 2 are presented in table 3. The three intersectional strata with highest risk of SGA included (i) <25-year-old immigrants with low education who were unmarried (OR 3.06, 95% CI 2.59–3.63), (ii) <25-year-old immigrants with low education who were married (OR 2.79, 95% CI 2.40–3.24) and (iii) 25- to 34-year-old immigrants with low education who were unmarried (OR 2.43, 95% CI 2.07–2.84). The three strata with the lowest risk of SGA included (i) women aged 25–34 years with high education born in Sweden who were married (Reference group), (ii) women older than 35 years born in Sweden with high education who were married (OR 1.01, 95% CI 0.90–1.13) and (iii) women aged 35 years or older with middle education who were born in Sweden and married (OR 1.14, 95% CI 0.97–1.35).

**Table 3:** ORs and 95% CIs from intersectional logistic regression analyses with all combinations of age, education, country of birth and civil status as the explanatory variables and SGA as the outcome

Age			Education			Migration status		Civil status		OR (95% CI)	Persons per stratum	SGA (%)
<25	25–34	≥35	Low	Middle	High	Immigrant	Born in Sweden	Married	Unmarried			
✔			✔			✔			✔	3.06 (2.59–3.63)	4031	154 (3.8)
✔			✔			✔		✔		2.79 (2.40–3.24)	5841	204 (3.5)
✔			✔				✔		✔	1.88 (1.69–2.09)	20 109	479 (2.4)
✔			✔				✔	✔		1.66 (1.30–2.11)	3331	70 (2.1)
✔				✔		✔			✔	2.23 (1.80–2.77)	3233	91 (2.8)
✔				✔		✔		✔		2.27 (1.93–2.68)	5702	163 (2.9)
✔				✔			✔		✔	1.27 (1.16–1.39)	49 361	801 (1.6)
✔				✔			✔	✔		1.26 (1.07–1.49)	1024	165 (16.1)
✔					✔	✔			✔	1.98 (1.29–3.03)	880	22 (2.5)
✔					✔	✔		✔		2.02 (1.59–2.58)	2739	70 (2.6)
✔					✔		✔		✔	1.18 (1.00–1.40)	10 008	151 (1.5)
✔					✔		✔	✔		1.26 (0.98–1.61)	4245	68 (1.6)
	✔		✔			✔			✔	2.43 (2.07–2.84)	5934	181 (3.1)
	✔		✔			✔		✔		2.18 (1.94–2.46)	12 462	343 (2.8)
	✔		✔				✔		✔	1.71 (1.50–1.95)	12 394	269 (2.2)
	✔		✔				✔	✔		1.56 (1.28–1.89)	5567	110 (2.0)
	✔			✔		✔			✔	1.83 (1.58–2.12)	9122	211 (2.3)
	✔			✔		✔		✔		1.68 (1.49–1.88)	17 715	377 (2.1)
	✔			✔			✔		✔	1.26 (1.17–1.36)	81 895	1.317 (0.0)
	✔			✔			✔	✔		1.16 (1.05–1.28)	4085	604 (14.8)
	✔				✔	✔			✔	1.66 (1.44–1.90)	11 378	239 (2.1)
	✔				✔	✔		✔		1.52 (1.38–1.67)	33 544	648 (1.9)
	✔				✔		✔		✔	1.18 (1.10–1.27)	109 887	1661 (1.51)
	✔				✔		✔	✔		REF	102 077	1306 (1.28)
		✔	✔			✔			✔	2.06 (1.53–2.77)	1773	46 (2.6)
		✔	✔			✔		✔		2.09 (1.70–2.57)	3791	100 (2.6)
		✔	✔				✔		✔	2.39 (1.84–3.12)	1962	59 (3.0)
		✔	✔				✔	✔		1.34 (0.86–2.09)	1175	20 (1.7)
		✔		✔		✔			✔	1.83 (1.42–2.36)	2762	64 (2.3)
		✔		✔		✔		✔		2.10 (1.74–2.52)	4839	128 (2.6)
		✔		✔			✔		✔	1.87 (1.66–2.11)	15 234	361 (2.4)
		✔		✔			✔	✔		1.14 (0.97–1.35)	10 923	159 (1.5)
		✔			✔	✔			✔	1.85 (1.51–2.26)	4405	103 (2.3)
		✔			✔	✔		✔		1.72 (1.50–1.98)	10 716	234 (2.2)
		✔			✔		✔		✔	1.56 (1.41–1.72)	28 618	566 (2.0)
		✔			✔		✔	✔		1.01 (0.90–1.13)	30 951	400 (1.3)

Note: Analyses performed among 679 694 women with singleton pregnancies giving birth in Sweden between 2010 and 2016.

The color shades represents the different sociodemographic factor is marked by each tick mark.

The AUC value increased from 0.567 (95% CI 0.562–0.573) in model 1 to 0.571 (95% CI 0.565–0.576) in model 2. Since CIs of the AUC values of models 1 and 2 were overlapping, no intersectional interaction in the multiplicative scale was detected. The AUC of both unidimensional model 1 and intersectional model 2 can be classified as very small/absent.

## Discussion

### Main findings

In this study, we adopted an intersectional approach to quantitatively investigate sociodemographic inequalities in the risk of SGA in Sweden. Our analyses show that education, age, country of birth and civil status all are individually associated with the risk of SGA. However, the intersectional model reveals a complex pattern and heterogeneities in SGA risk across the multi-dimensional intersections of the sociodemographic groups. For example, high education generally reduces risk of SGA. Despite this, the young immigrant women who have a high education nevertheless have rather high odds of SGA, indicating that a high education is less protective in this group. The highest AUC value obtained was 0.570, for the intersectional model, implying a very small DA of sociodemographic factors to identify SGA pregnancies and a large remaining heterogeneity within intersectional groups.

### Relation to previous studies

Comparisons of associations between sociodemographic exposures and different health outcomes must be interpreted with caution when studies are based on different populations. Having said that, our findings regarding the association between SGA and being an immigrant resemble the results from the UK when studying the risk of SGA in minority ethnic groups.^19^ Results are also in line with a previous study on adverse birth outcomes among different sociodemographic groups.^20^ Similar sociodemographic disparities have also been reported in unidimensional^21^^,^^22^ and intersectional studies on low birth weight.^23^

A previous study using Swedish data that assessed the DA of classical risk factors for SGA such as hypertension, smoking and previous SGA child found AUC values of similar magnitude (from AUC=0.50 for hypertension to 0.60 for smoking during pregnancy) as those reported in this study.^24^ Our results confirm their findings regarding how SGA is associated with low education status, relationship status and being born outside Sweden.

This is the first study to present a low DA to identify SGA pregnancies of an intersectional model that simultaneously considers several sociodemographic factors. Our finding on the heterogeneity in risk of SGA within groups defined by educational status or country of birth has not been described before. The absence of conclusive (multiplicative) intersectional interaction for SGA is also a novel finding.

The definition of SGA is uniform in this study, without considering sociodemographic factors. Some previous research has proposed to use customized SGA definitions that take certain maternal characteristics, including ethnicity, into account. This is based on findings of improved prediction of perinatal morbidity with customized SGA definitions.^24^ Other scholars have argued against customized SGA definitions, since heterogeneity within ethnic groups is larger than disparities between different ethnic groups, such customization would not improve clinical outcomes.^25^ The idea of offering growth assessment to specific women at increased risk of SGA has been evaluated in a randomized controlled trial comparing standard care (i.e. no ethnic targeting) with a protocol of growth assessment based on both clinical information and ethnicity. The trial found no effect on antenatal detection of SGA.^26^ This is in line with what would be expected based on the very small DA observed when taking the intersectionality between sociodemographic factors into consideration.

### Strengths and limitations

The study is based on high-quality register data and adopts a novel approach to the study sociodemographic disparities in risk of SGA. Additionally, all the variables used in our intersectional matrix are readily available in the clinical setting. The intersectional approach reveals heterogeneities within social categorization and the assessment of AUC cautions against too far-reaching conclusions based on measurements of average risk differences only.

With the cross-sectional observational design adopted, we studied associations rather than causal links. The sociodemographic exposures in our study are associated with several factors, that are in turn, associated with risk of SGA, such as comorbidities, smoking, and BMI. The sociodemographic factors are upstream compared with the above-mentioned factors, therefore those are considered as mediators rather than confounders. Adjusting for comorbidities, smoking and BMI would lead to an underestimation of the total effect of sociodemographic factors on the risk of SGA.

Almost all pregnancies that were excluded due to missing information lacked data on education. In this study population, missing information on education was frequent among immigrants. Statistics Sweden confirms that missing information on education is primarily an issue for immigrants with educational achievements outside Sweden.^14^ Statistics Sweden has collected data on education status through questionnaires distributed to immigrants, where the response rates are generally higher among people with higher education. Therefore, it is possible that the excluded pregnancies comprise immigrant women with low education to a larger extent and the exclusion of these pregnancies leads to an underestimation of socioeconomic disparities in risk of SGA in our study. When incorporating intersectional perspectives into public health, categorizations are inevitable. A limitation of our study is both that we exclude many sociodemographic factors that could be of interest and that we use few categories in order to not end up with too many intersectional strata and potentially few SGA cases in some of them. The categorization of women into married and unmarried does not consider the fact that today many couples live together without being married. Despite this, we consider marital status a proxy for a stable relationship that may be of importance for the risk of pregnancy complications. Similarly, grouping women into immigrants and people born in Sweden leads to a large heterogeneity in the immigrant category.

Efforts are made to eliminate race-based medicine and substitute it with race-conscious medicine.^27^ In Sweden, information on race/ethnicity is not registered in medical records nor legal to keep in population registers. Country of birth has recently been incorporated into antenatal clinical guidelines as a risk factor that should lead to consideration of thromboprophylaxis among pregnant women with mild COVID-19^5^ and induction of labour in late-term pregnancy.^6^ Intersectionality focuses on how power structures like gender, capitalism, racism and heteronormativity influence health. Therefore, we should ideally assess how the experience of racism influences health. Such information is not available in the registers used for this study. Nevertheless, we consider migration status a crude proxy for the experience of being an immigrant that is valuable to include in the intersectional matrix.

The frequency of SGA is 1.76%, which is lower than the expected 2.3% based on the definition of SGA of −2 SD used in our study. The exclusion of stillbirth pregnancies contributes to the low SGA prevalence, since the proportion of SGA children is higher among stillbirths than among live births. We also excluded pregnancies with birthweights out of range, it is plausible that a proportion of the excluded pregnancies represented true SGA children. In validation of MBR, Cnattingius et al. found that mistakes in data entry do occur and may be of specific importance when studying SGA. Therefore, we follow their procedure and exclude birth weights out of range.^28^

### Implications and future research

This study does not intend to provide a prediction model to guide public health nor clinical interventions, such studies require other research procedures.^29^ The welfare system in Sweden is characterized by universal rather than targeted interventions.^30^ According to the concept of proportionate universalism,^31^ some degree of targeting within a universal health care system is motivated if risk is concentrated to a specific part of the population. Our results call for further debate and research on the appropriateness of targeting individual-level medical interventions towards specific social groups defined in a unidimensional or categorical intersectional way. Intersectional analyses are still rather novel in public health so their implications for public health policy are not yet elaborated. With the evolution of precision medicine, interest in intersectional analyses may increase since intersectional analyses describe the social distribution of health hazards with a higher precision. In principle, we do not oppose the adoption of socially targeted medical interventions in antenatal care if it is justified by solid evidence. Whenever considering targeted care, statistical methods reporting quantifications of the DA constitute a litmus test of the relevance of the considered categories and should always be performed.^17^^,^^32^ When DA is small or very small, targeted interventions should not be recommended.

Whereas for human public health workers, the complexities arising from intersectional models may make such models impractical in a clinical setting, with increased use of computerized medicine and artificial intelligence, this problem will diminish.

A question that is implicitly approached in this study is if fetal growth assessment should be routinely offered to women with certain sociodemographic risk factors for SGA. In Sweden, some private antenatal care providers offer all women growth assessment whereas in public health care this is only performed on indication. Identification of pregnancies with a high risk of SGA is based on risk factors in the medical history, such as continuous smoking during pregnancy, low BMI, advanced age, chronic hypertension and previous pregnancies with FGR.^33^^,^^34^ Sociodemographic factors are not considered indications for growth assessment. The low DA we observe indicates that sociodemographic factors are a blunt tool to single out individuals for growth assessment and that such targeted interventions would not improve the identification of SGA pregnancies much. In addition to this, it remains unknown how women who belong to the groups we identified that had increased average risk of SGA would perceive targeted ultrasounds; either welcome it as improved access to antenatal care or consider it a stigma. This question should be investigated, preferably through qualitative studies involving the target population.

## Conclusion

The intersectional approach reveals a complex sociodemographic pattern of SGA risk. Within categories defined by only education status or country of birth, there are differences in risk that are attributable to the other sociodemographic factors of an individual. The very small AUC values in our study suggest that sociodemographic factors have a low accuracy when it comes to identifying individuals with an increased risk of SGA.

## Data Availability

The data that this study is based on are available from the Swedish National Board of Health and Welfare, and Statistics Sweden. However, register data are protected by rules of confidentiality. Researchers can only access the data after a special review that includes ethical approval from the authorities that control the data and from regional Ethics Committees. Swedish authorities do not provide individual-level data to researchers based in other countries. Researchers in other countries are instead advised to collaborate with Swedish colleagues in order to gain access to data. Key points Sociodemographic inequalities in the risk of SGA exist in Sweden. The risk of SGA among unmarried immigrant women under 25 with low education was a threefold higher compared to married, highly educated, native-born women aged 25 to 34. The SGA risk within groups defined by one social dimension is heterogenous, as shown in the intersectional analysis. Sociodemographic factors have a very small discriminatory accuracy to identify SGA cases at the individual level.
